# A Systematic Review and Meta-Analysis of Free Triiodothyronine (FT3) Levels in Humans Depending on Seasonal Air Temperature Changes: Is the Variation in FT3 Levels Related to Nonshivering Thermogenesis?

**DOI:** 10.3390/ijms241814052

**Published:** 2023-09-13

**Authors:** Alena A. Nikanorova, Nikolay A. Barashkov, Vera G. Pshennikova, Fedor M. Teryutin, Sergey S. Nakhodkin, Aisen V. Solovyev, Georgii P. Romanov, Tatiana E. Burtseva, Sardana A. Fedorova

**Affiliations:** 1 Yakut Science Centre of Complex Medical Problems, Yaroslavskogo 6/3, 677000 Yakutsk, Russia; nikanorova.alena@mail.ru (A.A.N.); psennikovavera@mail.ru (V.G.P.); rest26@mail.ru (F.M.T.); bourtsevat@yandex.ru (T.E.B.); sardaanafedorova@mail.ru (S.A.F.); 2 M.K. Ammosov North-Eastern Federal University, Kulakovskogo 46, 677013 Yakutsk, Russia; sergnahod@mail.ru (S.S.N.); nelloann@mail.ru (A.V.S.); gpromanov@gmail.com (G.P.R.)

**Keywords:** free triiodothyronine (FT3), seasonal variations, air temperature, polar T3 syndrome, nonshivering thermogenesis, brown adipose tissue (BAT), uncoupling protein 1 (UCP1)

## Abstract

Thyroid hormones play a crucial role in regulating normal development, growth, and metabolic function. However, the controversy surrounding seasonal changes in free triiodothyronine (FT3) levels remains unresolved. Therefore, the aim of this study was to conduct a systematic review and meta-analysis of variations in FT3 levels in relation to seasonal air temperatures in the context of current knowledge about its role in nonshivering thermogenesis. Ten eligible articles with a total of 336,755 participants were included in the meta-analysis. The studies were categorized into two groups based on the air temperature: “Cold winter”, where the winter temperature fell below 0 °C, and “Warm winter”, where the winter temperature was above 0 °C. The analysis revealed that in cold regions, FT3 levels decreased in winter compared to summer (I^2^ = 57%, *p* < 0.001), whereas in warm regions, FT3 levels increased during winter (I^2^ = 28%, *p* < 0.001). These findings suggest that seasonal variations in FT3 levels are likely to be influenced by the winter temperature. Considering the important role of the FT3 in the nonshivering thermogenesis process, we assume that this observed pattern is probably related to the differences in use of thyroid hormones in the brown adipose tissue during adaptive thermogenesis, which may depend on intensity of cold exposure.

## 1. Introduction

Thyroid hormones thyroxine (T4) and triiodothyronine (T3) play an important role in the processes of normal development, growth, and metabolic regulation [1,2]. T3 is more active than T4, though T4 constitutes more than 80% of the total volume of hormones released by the thyroid gland [3,4,5]. Therefore, the majority of T3 is produced extrathyroidally by the 5’-deiodination of the outer ring of T4 by deiodinase enzymes 1 and 2 [6]. The synthesis and secretion of thyroid hormones are modulated by the hypothalamic-pituitary-thyroid axis with the help of thyrotropin (TSH) [7]. To date, there are many studies devoted to the seasonal fluctuation of pituitary-thyroid hormones in adults. Multiple studies have shown that TSH levels were higher in winter than during other seasons in both euthyroid individuals and patients with various thyroid diseases [8,9,10,11,12,13,14,15,16,17,18]. In addition, the studies using large-scale data on TSH levels have also shown a seasonal dependence of TSH levels, with a peak in the winter [19,20].

However, for free triiodothyronine (FT3), it is difficult to trace seasonal dynamics as there are conflicting results of studies investigating seasonal changes in blood FT3 levels. A decrease in FT3 levels in winter compared to summer was first noted in employees of McMurdo stations in Antarctica [21,22,23]. This thyroid reaction is known as polar T3 syndrome [21]. In contrast, other studies have found that levels of FT3 rise in winter, and fall in summer [10,15,20,21,22,23,24,25,26]. However, there are also studies in which no significant differences in FT3 levels between winter and summer were found [12,27,28,29]. In addition, no changes in FT3 levels were observed in the studies including off-season between winter-spring and summer-autumn periods [14,30,31]. Thus, to date, there are conflicting views on seasonal variations in FT3 levels.

Therefore, the aim of this study is to conduct a systematic review and meta-analysis of variations in FT3 levels in relation to seasonal air temperatures in the context of current knowledge about its role in nonshivering thermogenesis.

## 2. Methods

### 2.1. Sources of Information and Search Strategy

The systematic review and meta-analysis were conducted in accordance with the PRISMA guidelines [32]. The methodological protocol of this systematic review and meta-analysis was registered in the International Prospective Register of Systematic Reviews PROSPERO (www.crd.york.ac.uk/PROSPERO, registration number ID CRD42023452291; accessed on 17 August 2023). The search for publications was conducted using the PubMed and Scopus databases. Classical articles, clinical studies, comparatives studies, and reviews were studied. Encyclopedia, book chapters, conference abstracts, case reports, conference info, correspondence, data articles, discussion, editorials, errata, examinations, mini reviews, patent reports, and short communications were excluded. The main search was performed from January to May 2023. PubMed and Scopus databases were searched in English for the period 1980 to May 2023 using a combination of keywords. The search strategy was carried out in the individual databases through the use of keywords. All possible combinations of the following keywords were used: (1) “free triiodothyronine” and “seasonal”, (2) “FT3” and “seasonal”, (3) “Free T3” and “seasonal”, (4) “free triiodothyronine” and “temperature”, (5) “FT3” and “temperature”, (6) “Free T3” and “temperature”. To search in the Scopus database, an advanced search was used, where in the line “Title, abstract or keywords specified by the author”, all 6 possible combinations of keywords and phrases “Review articles” and “Research articles” (automation tools) were found.

### 2.2. Eligibility Criteria

The inclusion criteria for the studies were as follows: (1) the studies examined seasonal variations in FT3 levels in euthyroid healthy adults; (2) in accordance with the methodology of the study, seasonal changes in FT3 levels had to be statistically significant (*p* < 0.05); (3) studies in other languages are allowed. The exclusion criteria for the studies were (1) lack of sufficient information on the levels of FT3; (2) no data on seasonality; (3) age less than 18 years; (4) duplicated study.

### 2.3. Selection Process

Relevant studies were analyzed, and the following data were collected: (1) author’s name and year of publication; (2–3) country and region where the study was performed; (4) study design; (5) sample size; (6) mean age or age range; (7) sex; (8) the average air temperature in winter and summer; (9) FT3 levels in winter and summer. Data were extracted independently by two researchers, and after matching and validation, including compliance with the norm, they were used for meta-analysis. Data on the concentrations of the hormones were expressed as pmol/L for FT3. If other units of measure were used in a publication, they were converted accordingly using the online calculator http://unitslab.com/, accessed on 16 May 2023.

### 2.4. Quality Control

The methodological quality of included studies was evaluated using the Newcastle-Ottawa scale [33]. A total of 9 items were used in this form. A study with more than 7 points was defined as a high-quality study. Fair quality was determined for the study, which received 5–6 points, and low quality studies received less than 5 points (<5 points unacceptable).

### 2.5. Data Analysis

Meta-analysis was performed using RevMan 5.3 (Cochrane Collaboration) https://revman.cochrane.org/info, accessed on 16 May 2023. The heterogeneity of the studies included in the meta-analysis was assessed using the I^2^ criterion. In accordance with the recommendations of Borenstein et al. [34] and Higgins et al. [35], a random-effects model with mean and standard deviation (SD) was used. The overall results were assessed for statistical significance using the Z-test with a 95% confidence interval. Results were considered statistically significant at *p* < 0.05. The inverse variance test (mean difference) was used for subgroup comparisons, with a 95% confidence interval and a significance level of *p* < 0.05.

### 2.6. Subgroups

We divided data from included original articles by the climatic environment into “Cold winter” and “Warm winter” subgroups. The “Cold winter” group included studies where the winter air temperature fell below 0 °C, and the “Warm winter” group included studies where the winter air temperature was above 0 °C. We used archived data from Time and Date AS weather summaries (https://www.timeanddate.com, accessed on 16 May 2023) to determine air temperatures.

## 3. Results

### 3.1. Systematic Review and Meta-Analysis of Variations in FT3 Levels in Relation to Seasonal Air Temperatures

**Figure 1:**
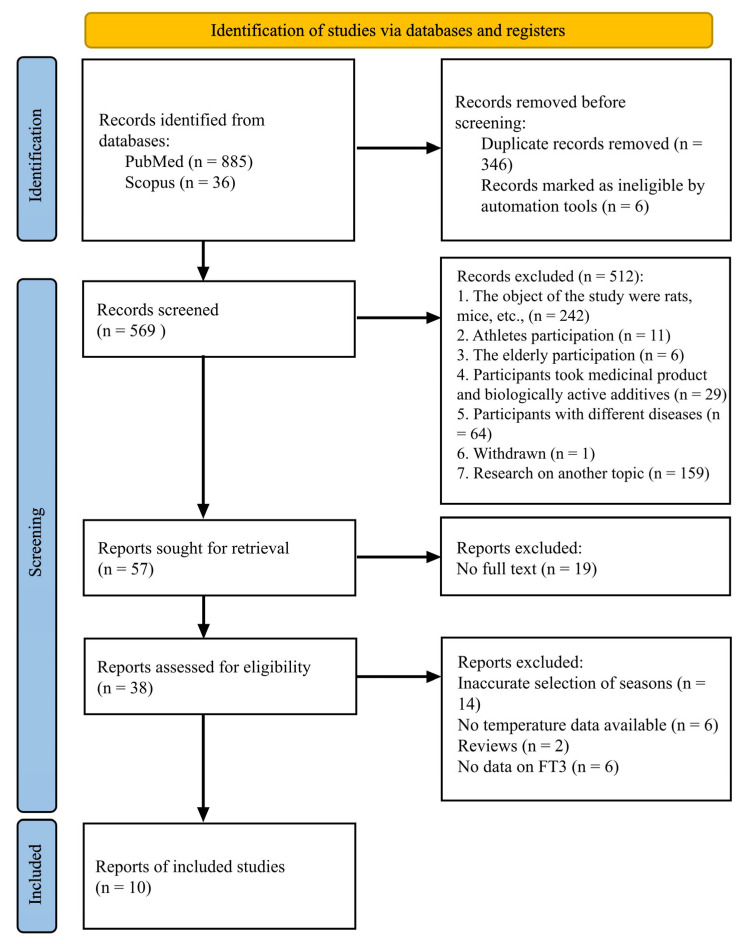
Flow diagram of the systematic review, presenting the study inclusion and exclusion process. **Note.** A representation of the process by which relevant studies were extracted from databases, selected, or excluded. Preferred reporting items for systematic reviews and meta-analyses (PRISMA) diagram for the study search [32].

We identified 921 potentially relevant studies from a PubMed (*n* = 885) and Scopus (*n* = 36) search. According to the flowchart, a total of 346 studies were excluded before screening as duplicate records (Figure 1). In addition, due to the use of filters only “Review articles” and “Research articles” in Scopus, 6 more publications were excluded. The remaining 569 studies were reviewed for titles and abstracts. Also excluded were studies where drugs and biologically active substances were used (*n* = 29), studies with patients suffering from various diseases (*n* = 64), studies with an elderly sample (*n* = 6) and with athletes (*n* = 11), and 159 publications were not related to the topic studied in this systematic review. The full texts of 57 articles were reviewed to assess their compliance with the inclusion criteria, and as a result, 47 more articles were excluded. As a result, 10 publications met the inclusion criteria and were included in the final analysis (Figure 1) [9,10,11,15,22,23,24,25,26,36].

The methodological quality of all included studies has been assessed using the Newcastle-Ottawa scale (<5 points unacceptable). Eight studies were of good quality (7–8 points) [9,10,11,15,24,25,26,36], and two were of fair quality (6 points) [22,23]. Research quality indicators, assessing the risk of bias, are presented in Supplementary Materials (Table S1). Among the included articles, six articles had a longitudinal study design and the other four had a cross-sectional study design. The analysis included a total of 336,755 individuals. Detailed characteristics of these studies are presented in Table 1.

#### Seasonal Variations of FT3 Levels Relation with Air Temperature, Depression, and Photoperiodism

**Figure 2:**
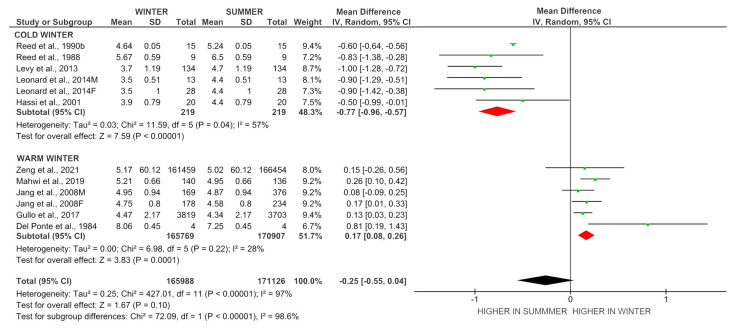
Forest plot of the FT3 levels in the groups “Cold winter” and “Warm winter” in winter and summer. **Note.** Reed et al., 1990b [22]; Reed et al., 1988 [23]; Levy et al., 2013 [11]; Leonard et al., 2014M [36]; Leonard et al., 2014F [36]; Hassi et al., 2001 [9]; Zeng et al., 2021 [15]; Mahwi et al., 2019 [26]; Jang et al., 2008M [10]; Jang et al., 2008F [10]; Gullo et al., 2017 [25]; Del Ponte et al., 1984 [24]; SD—standard deviation; CI—credible interval.

Figure 2 demonstrates the analysis of FT3 levels in winter and summer based on the region of residence. Individuals residing in the cold regions, where the winter temperature falls below 0 °C, exhibit lower FT3 levels in winter than in summer (I^2^ = 57%, *p* < 0.001). Conversely, those residing in the warm regions, where winter temperatures are above 0 °C, display higher FT3 levels in winter than in summer (I^2^ = 28%, *p* < 0.001). Previously, it was suggested that seasonal variations in FT3 levels can be influenced by photoperiodism and depression [21,22,37,38]. The studies that were included in the meta-analysis did not report the presence of any depressive disorders in their samples. Therefore, it can be assumed that depression can be excluded as a factor influencing the results obtained in this study. It seems that photoperiodism does not significantly affect seasonal variations in FT3. In this case, the level of FT3 should have been higher with longer daylight, and FT3 levels should be lower in winter than in summer. However, meta-analysis demonstrates that individuals living in warm regions with winter temperatures above 0 °C have higher serum FT3 levels during winter compared to summer (Figure 2), which is not consistent with photoperiodism. Thus, the results of this meta-analysis demonstrate that seasonal T3 levels correlate more with winter air temperature values than with depression and photoperiodism.

## 4. Discussion

### 4.1. The Dynamics of T3 Levels in Response to Cold Exposure

For the first time, changes in thyroid hormone levels in response to cold exposure were shown in people working at polar stations in Antarctica (MacMurdo, Zhongshan and the Great Wall) [12,21,22]. In the first similar study, a decrease in FT3 levels was recorded after a 42-week stay at the MacMurdo station, while no significant changes in FT4 and TSH levels were detected [21,22]. The study identified dynamics in the levels of hormones of the pituitary-thyroid system (in winter, the levels of FT3 decrease, the levels of TSH are normal/increase, FT4 are normal/decrease), referred to as “polar T3 syndrome” [21]. Subsequent studies have shown that signs of polar T3 syndrome are found outside Antarctica, among the indigenous inhabitants of Finland and Russia, where winter temperatures drop below −40 °C [9,36]. The study of the causes of the polar T3 syndrome in workers of polar stations showed that with prolonged exposure to cold, the rate of FT3 production and clearance from the blood increases, which means absorption of T3 by various tissues [21]. It is believed that changes in the dynamics of thyroid hormone levels in response to cold exposure begin with a decrease in FT3, followed by changes in FT4, total T3, and total T4 levels [8,9,39,40]. These alterations are considered to represent the principal features of a four-stage model of thyroid hormone adjustment during exposure to cold temperatures [40]. However, winter changes in thyroid hormone levels may not be significant, possibly depending on the duration and intensity of cold exposure on the individual and lifestyle factors [11,41,42]. For example, Inuit hunters from Greenland who were constantly exposed to cold had lower FT3 levels during winter than people living in more comfortable urban conditions [42]. Similar results were observed in Eastern Siberia among Yakut cattle breeders, where FT3, FT4, and TSH levels showed a more pronounced seasonal response in men who worked outside and were exposed to greater cold stress than men who worked in an office [11]. Also, studies on people acclimated to cold (ice-water swimming club) have shown that when exposed to cold, their FT3 levels decreased similarly to polar T3 syndrome, but no such changes were found in non-acclimated people [41]. The results of our meta-analysis demonstrate that in the cold regions with winter temperatures below 0 °C, FT3 levels are lower in winter than in summer, which is characteristic of the polar T3 syndrome (Figure 2). These findings confirm that signs of this syndrome extend to all regions with negative winter temperatures and are not limited to polar regions [11,41,42]. Thus, the analysis of T3 dynamics demonstrated a decrease in its levels in response to cold, and some authors explain it by higher absorption of T3 by tissues [11,21,42]. However, for a long time they could not determine which tissue absorbs so much T3 during cold exposure, until Anderson et al. [42] suggested that this may be required for brown adipose tissue (BAT). In this regard, we suggest that an increase in the absorption of T3 at the tissue level in winter may be associated with the activity of BAT and nonshivering thermogenesis.

### 4.2. T3 and Brown Adipose Tissue

Thyroid hormones play an important role in human thermoregulation; they regulate the rate of basal metabolism and participate in the processes of nonshivering thermogenesis. The involvement of thyroid hormones in thermoregulation processes has been noted for a long time. Since patients with hypothyroidism (the thyroid gland does not produce enough thyroid hormone) were intolerant of cold [43,44] and after treatment, sensitivity to cold was restored in humans [43,45]. It was later discovered that after treatment of hypothyroidism, the activity of BAT significantly increases in humans [45,46,47], which reinforces nonshivering thermogenesis [45]. It turned out that thyroid hormones are necessary to stimulate the activity of BAT [48,49,50,51]. The direct effect of T3 on mitochondrial autophagy, activity, and turnover in BAT, which are essential for thermogenesis, has been demonstrated on mouse models [52,53].

BAT is a thermogenic and highly plastic tissue that provides a longer and more stable source of heat by nonshivering thermogenesis. Previously, BAT was thought to be well developed only in newborns, but using positron emission tomography-computed tomography (PET-CT), active BAT was also found in adult humans [54,55,56,57,58]. BAT in an adult is localized mainly in the cervical, supraclavicular, paravertebral, periportal, and perinatal depots [55,57]. BAT specifically expresses the uncoupling protein-1, UCP1, which dissipates the proton gradient across the inner mitochondrial membrane, resulting in inefficiency during the formation of adenosine triphosphate (ATP) in oxidative phosphorylation. After the discovery that the thermogenesis of BAT is mediated by UCP1, subsequently the expression of *UCP1* and the UCP1 protein were usually used to describe the thermogenic ability of BAT [51]. The stimulation of BAT by thyroid hormones during nonshivering thermogenesis occurs by regulating the expression of the *UCP1* gene, but this requires increased concentrations of T3 [59,60,61]. These T3 concentrations are achieved when the activity of type 2 deiodinase (DIO2) in brown adipocytes via sympathetic nervous system stimulation leads to 5’-deiodination of the outer ring of T4, resulting in a three- to four-fold increase in T3 concentration [59,62,63,64]. This results in the saturation of thyroid hormone nuclear receptors—TR, leading to the activation of UCP1 and the full induction of BAT thermogenesis [51,53,62,65,66,67]. The BAT is present at birth and regresses with age, although it remains metabolically active in adulthood and/or may form from white adipocytes (browning) throughout life [68]. The browning is the transdifferentiation of white adipocytes into brown or beige adipose, which increases the cell thermogenic capacity [69,70,71,72]. However, another process was discovered—whitening of brown adipocytes, which occurs with physiological factors such as aging and obesity, which indicates the positive role of BAT for improving overall health [73,74,75]. In physiological conditions in adult humans, BAT and browning processes are activated by adrenergic stimuli (i.e., cold exposure), a high-fat diet, and physical exercises [47,48,49,50,51,52,53,54,55,76,77,78,79,80,81,82,83]. PET-CT showed the presence of active BAT in adult humans who were training for one-two hours in 12–17 °C cold [76]. Thus, thyroid hormones participate in modeling the function of BAT by regulating the expression of the *UCP1* gene in brown adipocytes through adrenergic stimuli predominantly under cold stress.

### 4.3. T3 and Adaptive and “Activated” Thermogenesis

Currently, it is believed that thyroid hormones can participate in the regulation of different nonshivering thermogenesis processes: under cold exposure named as adaptive thermogenesis [42,79,80] and under thermoneutral conditions named as “activated” thermogenesis [52,53]. Yau et al. demonstrate that thyroid hormones can stimulate the activity of BAT even without cold exposure in thermoneutral conditions, a process referred to as “activated” thermogenesis [52,53]. An important distinguishing feature (except temperature) is the occurrence of shiverings in combination with adaptive thermogenesis, whereas with activated thermogenesis, there are no shiverings [52,53]. For both types of thermogenesis, DIO2 is induced, which leads to an increase in intracellular T3 [52,53]. It has been shown that mice with DIO2 knockout, which, despite normal T3 levels and even elevated serum T4 levels, demonstrated impaired BAT function, and their inability to properly respond to the effects of cold decreases, which leads to hypothermia [59,84]. In adaptive thermogenesis, induction of serum T3 may occur, so the concentration of T3 in serum may be variable and may depend on temperature and duration of exposure to cold [52,53]. On the other hand, in the activated thermogenesis, there is a significant increase in the serum thyroid hormone concentration [52,53]. Thus, thyroid hormones can participate in modulating the function of BAT under different intensities of cold exposure and even thermoneutral conditions.

### 4.4. The T3 Stimulation of the Adaptive Thermogenesis Pathways under the Influence of Different Winter Temperature

Considering the current knowledge about the role of the FT3 in the nonshivering thermogenesis process, revealed patterns of the seasonal variation of FT3 levels can be explained by active BAT in adult humans in all regions of the world.

We suppose that regions of the world where the winter temperature fell below 0 °C activated the adaptive thermogenesis process. In this process, BAT uses more T3 as intracellular and serum to activate full induction nonshivering thermogenesis, resulting in decreased levels of serum T3 in the blood. This finding explains the signs of the polar T3 syndrome in the residents of the coldest regions of the world. In addition, this assumption is supported by the data of molecular-genetic analysis related to human adaptation to a cold climate, where the increased prevalence of the T-allele-rs3811787 of the *UCP1* gene associated with a more active form of UCP1 that may use more T3 has been found in northern Eurasia, along the shore of the Arctic Ocean [85].

In the regions where the winter temperature was above 0 °C, it is more possible to have a less intense nonshivering thermogenesis (referred to by us as “mild” adaptive thermogenesis). In order to activate “mild” adaptive thermogenesis (close to thermoneutral conditions, which somewhat resemble the “activated” thermogenesis process demonstrated by Yau [52,53]) BAT, it is enough to use intracellular T3 and not take it additionally from the blood, resulting in an increase of serum T3 levels. This assumption is supported by the data, where it was shown that in euthyroid men and women, short-term mild cold exposure (from 1 to 22 °C, 2–8 h a day) increased the levels of FT3 [86,87]. A stronger short-term exposure to cold, 3 h a day from −40 °C to −10 °C, increased the serum levels of the FT3 and FT4 [88].

**Figure 3:**
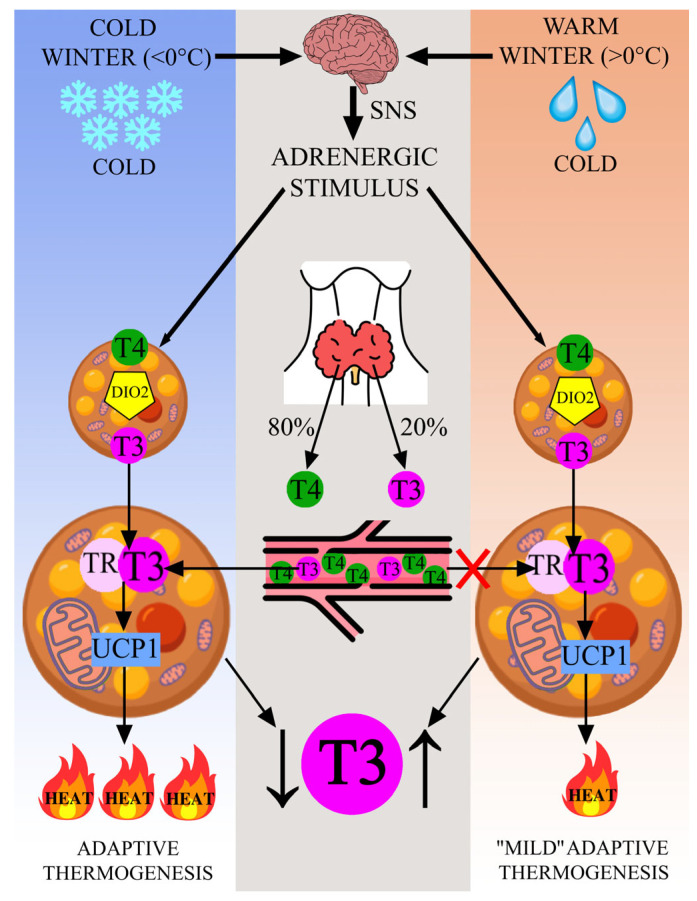
Stimulation of the adaptive thermogenesis pathways by the thyroid hormones under the influence of different winter temperatures. **Note**. *Adaptive thermogenesis*: severe cold in winter (air temperature is below 0 °C) activates the sympathetic nervous system (SNS), and an adrenergic stimulus increases the activity of type 2 deiodinase (DIO2) in BAT. The amounts of intracellular T3 increase in brown adipocytes and additionally use T3 from the blood. After interaction of T3 with the nuclear receptor—TR, the TR-T3 complex additionally increases the activity of UCP1 (uncoupling protein 1). There is a powerful release of heat and the level of serum T3 decreases. “*Mild*” *adaptive thermogenesis*: mild cold in winter (air temperature is above 0 °C) activates the SNS, and the adrenergic stimulus increases the activity of DIO2 in BAT. As a result, the amount of intracellular T3 increases in brown adipocytes but additionally not use T3 from the blood (red cross). After interaction of T3 with the nuclear receptor—TR, the TR-T3 complex additionally increases the activity of UCP1. Low-power heat generation occurs and the level of serum T3 increases.

In order to describe a potential T3-TR-UCP1 interaction pathway, we present the stimulation of adaptive thermogenesis and “mild” adaptive thermogenesis by the thyroid hormones under the influence of different winter temperatures in Figure 3.

### 4.5. The Regulation by the TSH of the T3 Levels under the Adaptive and “Mild” Adaptive Thermogenesis

The difference between adaptive thermogenesis and “mild” adaptive thermogenesis is the change in T3 levels in the blood. During exposure to cold, the active BAT uses T3 to induce adaptive thermogenesis. At that time, T3 levels in brown adipocytes concentration increase three- to four-fold due to the deiodination of T4 into T3 [59,61,62,63,64], and T3 levels in the blood do not change. An imbalance between intracellular and extracellular T3 levels occurs [88]. In turn, the pituitary gland tries to regulate the resulting imbalance by increasing TSH levels [88], which should lead to an increase in T3 levels in the blood. However, with strong and prolonged cold exposure, the BAT will consume a lot of T3, and it can take T3 directly from the blood. Therefore, in regions with “Cold winters”, it is possible to observe a tendency to decrease T3 levels in the blood in winter compared to summer and an inverse tendency for TSH levels [8,9,10,11,12,13,14,15,16,17,18,19,20]. In regions with “Warm winters”, a similar imbalance also occurs in response to moderate cold exposure. However, there is less active BAT, and accordingly it consumes less T3 and does not take T3 from the blood. Since the imbalance persists, the pituitary gland is also forced to increase TSH levels, which leads to an increase in T3 in the blood. Therefore, in regions with warm winters, there is a tendency to increase the levels of T3 and TSH in the blood in winter compared to summer.

Thus, our proposed concept that active BAT in adults in all regions of the world can affect blood T3 levels depending on the air temperature in winter (regions with “Cold winter” and “Warm winter”) may explain the contradictory results about seasonal variations of this hormone [10,12,15,20,21,22,23,24,25,26,27,28,29,30,31] and it is consistent with the previously obtained results on the seasonal dynamics of TSH levels [8,9,10,11,12,13,14,15,16,17,18,19,20].

### 4.6. The Relationship of Thyroid Hormones with Basal Metabolic Rate under Cold Exposure

Many studies of indigenous populations living in cold climates note that they have a higher basal metabolic rate (BMR) compared to international standards which is considered as an adaptation of the body to conditions of chronic and severe cold stress by increasing of heat production [39,89,90,91,92,93]. Thyroid hormones are suggested to be involved in the mechanisms of BMR increase in winter, since they play an important role in modulating BMR and the rate of energy expenditure at rest [11]. The relationship between seasonal fluctuations in thyroid hormones and seasonal changes in BMR has been shown in several studies [36,37,94]. One consistent pattern observed is a decrease in FT3 levels and an increase in BMR during winter. Several studies have shown that BMR has seasonal dynamics, with BMR increasing in winter and decreasing in summer [36,37,94]. However, these seasonal changes are highly dependent on parameters such as cold exposure and physical activity. Thus, physical inactivity during expeditions in Antarctica led to a decrease in BMR among military and scientific personnel [95,96,97,98,99]. In contrast, a study by Reed et al. [37], where employees of polar stations regularly engaged in outdoor activities, showed a significant increase in BMR in winter. Recent studies of the indigenous people of Eastern Siberia have shown that, despite the modern type of diet and hypodynamia during the long winter period, body composition and weight did not have seasonal dynamics, while thyroid hormone levels showed signs of polar T3 syndrome, which correlated with seasonal dynamics of the BMR changes [36]. It is possible that the absence of changes in the composition and body weight of the inhabitants of Eastern Siberia is associated with the presence of the BAT, the activity of which under the cold exposure can lead to a change in the clearance of thyroid hormones and an increase in BMR, which requires increased energy consumption and does not significantly affect the deposition of fat mass, despite changes in the traditional lifestyle and type of diet associated with global urbanization.

### 4.7. Perspective and Further Study

According to the World Health Organization, obesity is defined as abnormal or excessive fat accumulation and is pertaining to adults, adolescents, and children worldwide [98,99]. In 2016, over 650 million adults were obese, representing 13% of the adult population [98,99]. In the past ten years, a special studies focus has been put on the mechanisms underlying heat generation, as these processes could theoretically be used to fight obesity and metabolic disorders. Thus, BAT constitutes a potential anti-obesity drug target [51,100,101]. In addition, the structure of the human protein UCP1 has recently been obtained, which can help in the development of drugs for obesity and various complications associated with metabolism [102]. Currently, it is known that there are various ways to activate BAT: physiological, natural, and synthetic (pharmacological) [100,101]. Physiological means activation of BAT under certain environmental conditions (for example, cold) [54,56,58,103,104]. Natural activation of BAT assumes effects of substances of natural origin such as menthol, capsaicin, berberine, resveratrol, quercetin, baykalein, and many others [105,106,107,108,109,110,111,112]. For example, menthol and capsaicin are able to increase the expression of UCP1 through the TRP channel (TRPM8 and TRPV1) [109,110,111]. Synthetic (pharmaceutical) activators of BAT include small molecules that act on the central nervous system to activate thermogenic effects [113,114,115,116,117,118,119,120,121]. These include beta-3-adrenergic receptor agonists (isoproterenol, dobutamine, formoterol, CL316243, Rb1, mirabegron) [113,114,115,116,117], thyroid receptor agonists (levothyroxine, liothyronine) [118], and farnesoid X-receptor agonists (farnesol, fexeramine, CDCA) [119,120,121]. Since human and animal models may have different responses to the stimulation of the BAT [102], we suppose that the thyroid hormones interaction pathway may be one of the curious ways of BAT stimulation. Our findings of seasonal variations in FT3 levels suppose that this way is possibly more physiological for humans. However, the complex interplay of thyroid hormones with all these systems and their interactions among each other is far yet from being understood. Thus, a positive correlation with obesity in euthyroid individuals was found for serum TSH levels [122,123,124,125,126,127,128,129,130,131,132,133,134]. However, studies examining the relationship of FT3, FT4, and obesity have yielded conflicting results [123,124,126,131,133,134,135,136,137,138] and the reasons for these discrepancies remain unclear. Some researchers suggest that in obesity, an increase in TSH levels serves to maintain thyroid hormone levels within the euthyroid state. This is because with excess fat mass, as with cold exposure, the conversion rate of T4 to T3 increases along with the utilization of these hormones to enhance energy consumption [137,139]. This response of the hypothalamus-pituitary–thyroid axis is considered as an adaptive compensatory mechanism in reaction to obesity, aimed at maintaining or reducing weight [137,139]. Our results on the seasonal dynamics of FT3 suggest that to understand the causes influencing thyroid hormones in obesity, one must consider not only factors like age, sex, different degrees of obesity, fat percentage, leptin, insulin sensitivity, smoking habits, iodine intake, and hypocaloric diet [124,125,130,135,136,140], but also environmental factors, specifically winter air temperature. We believe that further studies of the nonshivering thermogenesis process under thyroid hormones regulation have a good perspective and could help to search for ways of therapy of metabolic impairments.

### 4.8. Limitations of the Study

This study has some limitations. Although we assigned the delineator of winter temperature thresholds, which allowed us to discover the pattern of the difference seasonal variations of FT3 levels and proposed the concept that active BAT in adult humans can affect this seasonal variation, additional experimental data are required to confirm this dependence in the worldwide population. Furthermore, our research has certain limitations concerning details of the meta-analysis data set. Firstly, due to the lack of sufficient data, we were unable to conduct an analysis separately for females and males. Secondly: we neglected age indicators; people of different ages were included in the analysis.

## 5. Conclusions

In this study, for the first time we present systematic review and meta-analysis of seasonal variations of free Triiodothyronine (FT3) levels depending on seasons, which included 336,755 participants. Obtained results demonstrate that seasonal variations in FT3 levels are dependent on air temperature during winter. In cold regions of the world, where air temperature drops below 0 °C during winter, a decrease in FT3 levels can be observed, similar to the polar T3 syndrome. Conversely, in warm regions where air temperature remains above 0 °C, FT3 levels are higher in winter compared to summer. After systematic review of the current knowledge about the role of the FT3 in nonshivering the thermogenesis process, we conclude that this observed pattern is probably related to the differences in the use of thyroid hormones during adaptive thermogenesis, which can be influenced by environmental factors, particularly the intensity of cold exposure on the organism.

## Figures and Tables

**Table 1:** Characteristics of the studies included in the meta-analysis.

#	Author, Year	Country	Region	Study Design	Sample Size		Age, Years	Sex	Temperature, °C		FT3, pmol/L	
									Winter	Summer	Winter	Summer
**Cold Winter**												
1	Reed et al., 1988 [23]	USA and Antarctica	Los Angeles and McMurdo Station	L	9	25 ± 1.0		M	−20	+25	5.67 ± 0.51	6.5 ± 0.26
2	Reed et al., 1990b [22]	USA and Antarctica	Los Angeles and McMurdo Station	L	15	24.5 ± 0.9		M	−29	+22	4.64 ± 0.14	5.24 ± 0.18
3	Hassi et al., 2001 [9]	Finland	province of Lapland	L	20	26–40		M	−10	+15	3.9 ± 0.1	4.4 ± 0.2
4	Leonard et al., 2014 [36]	Russia	Republic of Sakha (Yakutia)	L	13	18–49		M	−34	+18	3.5 ± 0.19	4.4 ± 0.14
	Leonard et al., 2014 [36]	Russia	Republic of Sakha (Yakutia)	L	28	18–49		F	−34	+18	3.5 ± 0.7	4.4 ± 0.7
5	Levy et al., 2013 [11]	Russia	Republic of Sakha (Yakutia)	L	134	19–49		M/F	−34	+18	3.7 ± 1.5	4.7 ± 1.1
**Warm winter**												
6	Mahwi et al., 2019 [26]	Iraq	Kurdistan Region, Sulaymaniyah	C	140		34 ± 12	M/F	+7	+34	5.21 ± 0.05	4.95 ± 0.06
7	Zeng et al., 2021 [15]	China	Sichuan Province	C	327913		18–90	M/F	+5	+33	5.17 ± 0.001	5.02 ± 0.002
8	Jang et al., 2008 [10]	South Korea	Gyeongsang Province	C	545		18–65	M	+7	+28	4.95 ± 0.07	4.87 ± 0.04
	Jang et al., 2008 [10]	South Korea	Gyeongsang Province	C	412		18–65	F	+7	+28	4.75 ± 0.91	4.58 ± 0.77
9	Del Ponte et al., 1984 [24]	Italy	Province of Chieti	L	4		24–28	M	+9	+25	8.06 ± 0.38	7.25 ± 0.37
10	Gullo et al., 2017 [25]	Italy	City of Catania	C	7522		37–61	M/F	+10	+25	4.47 ± 0.54	4.34 ± 0.52

**Note.** L—Longitudinal study design, C—Cross-sectional study design; M—Male, F—Female.

## Data Availability

The data presented in this study are available on request from the corresponding author.

## Supplementary Materials

The following supporting information can be downloaded at: https://www.mdpi.com/article/10.3390/ijms241814052/s1.
