# Supplementary material for: A Systematic Review and Meta-Analysis of Free Triiodothyronine (FT3) Levels in Humans Depending on Seasonal Air Temperature Changes: Is the Variation in FT3 Levels Related to Nonshivering Thermogenesis?
Source: Int J Mol Sci. 2023 Sep 13;24(18):14052. doi: 10.3390/ijms241814052 (PMC10531421; doi:10.3390/ijms241814052)
Supplement: Supplementary file 1 [file ijms-24-14052-s001.zip › ijms-2597091-supplementary.pdf]

Supplementary materials

Table S1. Quality score of the included studies.

Study	Representativeness of the exposed cohort	Selection of the non-exposed cohort	Ascertainment of exposure	Demonstration that outcome of interest was not present at start of study	Compare ability of cohorts on the basis of the design or analysis	Assessment of outcome	Was follow up long enough for outcomes to occur	Adequacy of follow up of cohorts	Total score	Power
Reed et al., 1988			*	*	*	*	*	*	6*	Fair quality
Reed et al., 1990b			*	*	*	*	*	*	6*	Fair quality
Hassi et al., 2001	*	*	*	*	*	*	*	*	8*	Good quality
Leonard et al., 2014	*	*	*	*	*	*	*	*	8*	Good quality
Levy et al., 2013	*	*	*	*	*	*	*	*	8*	Good quality
Mahwi et al., 2019	*	*	*	*	*	*	*	*	8*	Good quality
Zeng et al., 2021	*	*	*	*	*	*	*	*	8*	Good quality
Jang et al., 2008	*	*	*	*	*	*	*	*	8*	Good quality
Del Ponte et al., 1984		*	*	*	*	*	*	*	7*	Good quality
Gullo et al., 2017	*	*	*	*	*	*	*	*	8*	Good quality

Note. * - one score.
